# Preventive Effects of Schisandrin A, A Bioactive Component of *Schisandra chinensis*, on Dexamethasone-Induced Muscle Atrophy

**DOI:** 10.3390/nu12051255

**Published:** 2020-04-28

**Authors:** MyeongHoon Yeon, Hojung Choi, Hee-Sook Jun

**Affiliations:** 1 College of Pharmacy and Gachon Institute of Pharmaceutical Science, Gachon University, 191 Hambakmoe-ro, Yeonsu-gu, Incheon 21936, Korea; 2 Lee Gil Ya Cancer and Diabetes Institute, Gachon University, 155 Gaetbeol-ro, Yeonsu-gu, Incheon 21999, Korea; 3 Gachon Medical Research Institute, Gil Hospital, 21 Namdong-daero774beon-gil, Namdong-gu, Incheon 21565, Korea

**Keywords:** *Schisandra chinensis*, Schisandrin A, muscle atrophy, protein degradation, protein synthesis

## Abstract

Muscle wasting is caused by various factors, such as aging, cancer, diabetes, and chronic kidney disease, and significantly decreases the quality of life. However, therapeutic interventions for muscle atrophy have not yet been well-developed. In this study, we investigated the effects of schisandrin A (SNA), a component extracted from the fruits of *Schisandra chinensis*, on dexamethasone (DEX)-induced muscle atrophy in mice and studied the underlying mechanisms. DEX+SNA-treated mice had significantly increased grip strength, muscle weight, and muscle fiber size compared with DEX+vehicle-treated mice. In addition, SNA treatment significantly reduced the expression of muscle degradation factors such as myostatin, MAFbx (atrogin1), and muscle RING-finger protein-1 (MuRF1) and enhanced the expression of myosin heavy chain (MyHC) compared to the vehicle. In vitro studies using differentiated C2C12 myotubes also showed that SNA treatment decreased the expression of muscle degradation factors induced by dexamethasone and increased protein synthesis and expression of MyHCs by regulation of Akt/FoxO and Akt/70S6K pathways, respectively. These results suggest that SNA reduces protein degradation and increases protein synthesis in the muscle, contributing to the amelioration of dexamethasone-induced muscle atrophy and may be a potential candidate for the prevention and treatment of muscle atrophy.

## 1. Introduction

Skeletal muscle atrophy is caused by several factors including genetic factors, various diseases, disuse, and aging [1]. As the lifespan of humans increases with the development of medicine, age-associated skeletal muscle atrophies are becoming a global social problem due to deterioration of the quality of life [2]. Nowadays, many studies are being conducted to develop therapeutic drugs for the treatment of muscle atrophy. However, aside from exercise regimens, there are currently no effective medicines or therapeutic options available.

In general, the maintenance of muscle mass depends on the balance between protein degradation and synthesis, and both these processes are sensitive to factors such as hormonal balance, nutritional status, physical activity/exercise, injury, and disease [3]. Muscle protein degradation occurs through the ubiquitin-proteasome-dependent pathway, caspase system pathway, and autophagy pathway [4]. Of these, the ubiquitin-proteasome system (UPS) mediates the degradation of short-lived proteins and has been indicated as an important mechanism influencing muscle atrophy [5]. Two major muscle-specific ubiquitin E3 ligases that contribute to the ubiquitination process include muscle RING-finger protein-1 (MuRF1) and MAFbx (atrogin1).

Various animal models have been used in muscle atrophy studies such as hindlimb suspension, casting, starvation, denervation, diabetes, and the administration of glucocorticoid (GC) [6,7]. GC administration is a widely used animal model for the study of catabolic muscle atrophy [8]. The administration of dexamethasone (DEX) in high concentrations stimulates muscle proteolysis [8]. In general, plasma GC levels increase due to altered neuroendocrine regulation. GC is involved in various catabolic systems in muscles, connective tissues, bones, and lymphoid tissues [9]. GC is used to treat a variety of disease states, but long-term use or administration of high doses can upregulate myostatin expression and increase the expression of E3 ubiquitin ligase, atrogin1, and MuRF1, eventually leading to muscle atrophy [10,11].

*Schisandra chinensis* is a herb traditionally used in Korea and China and is cultivated in northwest China, Korea, and eastern Russia. Modern pharmacological studies have proven that *S. chinensis* has anti-inflammatory, anti-hepatotoxic, and anti-nephrotoxic effects [12,13,14]. In addition, recent studies have shown that the *S. chinensis* extract alleviates DEX-, neurectomy-, and disuse-induced muscle atrophy [8,15,16]. However, not much is known about the effects of the active ingredient of the *S. chinensis* extract with respect to improving muscle atrophy. Many dibenzocyclooctadiene lignans have been isolated from the fruits of *S. chinensis,* including schisandrin (SLA, schisandrol A), schisandrin B (SLB, gomisin N), γ-schisandrin, schisantherin A (SA, gomisin C), deoxyschisandrin (SNA, schisandrin A), and gomisin A [17]. SNA is one of the major lignans found in the fruits of *S. chinensis* [18,19]. In this study, we investigated the effects of SNA on DEX-induced muscle atrophy in vivo and studied the mechanisms involved, particularly in muscle protein degradation and synthesis.

## 2. Materials and Methods

### 2.1. Animals

Eight-week-old C57BL/6 male mice were obtained from Orient Bio (Seongnam-si, Kyunggido, Korea) and allowed 1 week of adaptation before the study. The mice were also adapted towards oral administration for 1 week. Mice were maintained under 23 ± 1 °C with 12 h light/dark cycles with free access to water and a regular chow diet. All animal experiments were performed in compliance with the ethical requirements of the Laboratory Animal Research Center, College of Pharmacy, Gachon University. The experimental protocol was approved by the Gachon University Institutional Animal Care and Use Committee (GIACUC-R2018012).

### 2.2. Induction of Muscle Atrophy and Treatment with SNA

C57BL/6 male mice (10-week-old) were randomly divided into three groups: control (CON), dexamethasone (DEX; D4902, Sigma-Aldrich, MO, USA), and dexamethasone + schisandrin A (DEX + SNA) groups. CON was administered an intraperitoneal (i.p.) injection of 9% Kolliphor^®^ HS 15 (42966, Sigma-Aldrich, MO, USA) + 10% DMSO (D1370, Duchefa Biochemie, BV, Netherlands) and orally administered 0.5% carboxymethyl cellulose sodium (CMC, C0045, TCI, Tokyo, Japan). The DEX group was administered an i.p. injection of DEX dissolved in 9% Kolliphor^®^ HS 15 + 10% DMSO solution and orally administered 0.5% CMC for 8 days. The DEX + SNA group was orally administered SNA (C3501 TCI, Tokyo, Japan) 20 mg/kg in 0.5% CMC once a day for 10 days, after 2 days DEX dissolved in 9% Kolliphor^®^ HS 15 + 10% DMSO solution was injected i.p. for 8 days until the end of experiment. On day 10, the grip strength of the mice was tested to measure the muscle force. The mice were then sacrificed for further skeletal muscle tissue analysis. During the course of the experiment, the body weight and food intake of the mice were checked daily between 1:00–2:00 p.m.

### 2.3. Cell Culture

The C2C12 cells (CRL-1772, ATCC^®^, USA) were grown in Dulbecco’s modified Eagle’s medium (LM001-05, Welgene, Gyeongsangbuk-do, Korea) supplemented with 10% fetal bovine serum (S001-07, Welgene, Gyeongsangbuk-do, Korea), 0.2 mM glutamine, 100 IU/mL penicillin, and 0.1 mg/mL streptomycin. Cells were cultured in 5% CO_2_ at 37 °C. To differentiate C2C12 myoblasts from C2C12 myotubes, C2C12 cells were seeded at 2.5 × 105 cells per 6-well plate, and the medium was then replaced with a differential medium containing 2% horse serum (16050-122, Thermo Fisher scientific, MA, USA), 100 IU/mL penicillin, and 0.1 mg/mL streptomycin for five days. The C2C12 myotubes were treated with 10 μM SNA and 1 μM DEX for 12 h. To further investigate the molecular mechanism of SNA action, C2C12 myotubes were treated with 20 μM SNA and 10 μM DEX for 24 h.

### 2.4. Measurement of Grip Strength

After 10 days of SNA administration, mice were subjected to grip strength analysis to measure the muscle force. Limb grip strength was measured using a grip strength meter (BIO-G53, BIOSEB, FL, USA). Briefly, to assess forelimb strength, mice were allowed to rest on a T-bar such that they could tightly grip the T-bar using only the two forelimbs. The tail of each mouse was pulled directly toward the tester and parallel to the T bar with the same force. Grip strength was calculated as force divided by the final body weight (N/g).

### 2.5. Tissue Collection

After the mice were sacrificed, tissues were quickly excised, carefully dissected, and weighed. The skeletal muscles isolated were quadriceps femoris (QD), gastrocnemius (GA), extensor digitorum longus (EDL), soleus (SOL), and tibialis anterior (TA). Muscle samples were stored at −80 °C until further analyses could be performed.

### 2.6. Histology

TA muscles were fixed in 10% neutral buffered formalin and embedded in paraffin. These paraffin blocks were cut into 4 µm thick sections and stained with hematoxylin (30002, Muto Pure Chemicals Co., Ltd., Japan) and eosin (HT110132, Sigma-Aldrich, MO, USA) (H&E). The H&E-stained sections were used for the cross-sectional area (CSA) analyses. These sections were examined (200 × magnification) using a confocal microscope (Nikon intensilight C-HGFI, Tokyo, Japan) and NIS-element AR 4.00.00 software. Then, the myofiber cross-sectional areas were analyzed using the ImageJ software.

### 2.7. Real-Time Polymerase Chain Reaction (PCR) Analysis

Total RNA was isolated from the TA muscle using RNAiso Plus (9108, TAKARA, Kyoto, Japan) according to the manufacturer’s protocol. Complementary DNA (cDNA) was synthesized from 2 μg total RNA with the PrimeScript 1st-strand cDNA synthesis kit (6110A, TAKARA, Kyoto, Japan). Quantitative real-time PCR (RT-qPCR) was performed using a reaction mixture containing SYBR™ Green master mix (RR820A, Takara, Kyoto, Japan). Results were calculated using the 2-ΔΔCT relative quantification method normalized to the Cyclophilin B gene. The sequences of the primer pairs are shown in Table 1.

### 2.8. Western Blot Analysis

Total protein from the TA muscle or C2C12 myotube was isolated with a mammalian protein extract buffer (78501, GE Healthcare Life Sciences, MA, USA) containing a protease inhibitor mixture (P8340, Sigma-Aldrich, MO, USA) and phosphatase inhibitors (P5726 and P0044, Sigma-Aldrich, MO, USA). Then, the isolated protein was separated by SDS-polyacrylamide gel electrophoresis. The transferred membrane was blocked with 5% skimmed milk or 5% BSA for 1 h and incubated with the following primary antibodies overnight: 1:1000 (anti-GDF8/myostatin (ab203076, Abcam, MA, USA), anti-MYH (B-5) (sc-376157, Santa Cruz biotechnology, CA, USA), anti-p-p70S6K (9205, Cell signaling, MA, USA), anti-p70S6K (9202, Cell signaling, MA, USA), anti-p-Akt (Ser473) (4060, Cell signaling, MA, USA), anti-Akt (9272, Cell signaling, MA, USA), anti-p-FoxO1 (Ser256) (9461, Cell signaling, MA, USA), anti-FoxO1 (2880, Cell signaling, MA, USA), 1:2000 (anti-MuRF1 (ab172479, Abcam, MA, USA), anti-MAFbx (sc-166806, Santa Cruz biotechnology, CA, USA)), and 1:5000 (anti-GAPDH (sc-32233, Santa Cruz biotechnology, CA, USA)). GAPDH was used as a loading control for the assay. The membrane was washed three times with TBST for 10 min and then incubated with horseradish peroxidase (HRP)-conjugated goat anti-rabbit IgG or goat anti-mouse IgG secondary antibodies. The target complex was detected by Chemidoc^TM^ XRS+ system with Image Lab^TM^ software (Bio-Rad, CA, USA) and the band intensity was quantified using the Image Lab program.

### 2.9. Surface Sensing of Translation (SUnSET) Assay

Protein synthesis was measured using the SUnSET technique, as previously described [20]. Briefly, C2C12 myoblasts were differentiated for 5 days. After 5 days, the cells were treated with or without 10 μM DEX and 20 μM SNA for 12 h, followed by 1 μM puromycin (P8833, Sigma-Aldrich, MO, USA) and incubated for 30 min. Puromycin-labelled proteins were assessed using western blotting with an anti-puromycin antibody (MABE343, Sigma-Aldrich, MO, USA) using an equal amount of total protein per sample.

### 2.10. Measurement of Myotube Diameter in C2C12 Myotubes

C2C12 myoblasts were differentiated for 5 days and then treated with or without 1 μM DEX and 10 μM SNA for 12 h. After 12 h, at least 5 different areas of each plate (3 plates per group) were photographed using a microscope. Then, the diameter of myotubes was measured and analyzed using ImageJ software.

### 2.11. Statistical Analysis

Data are presented as the mean ± standard error of the means (S.E.M) or standard deviation (S.D.). Statistical analysis was performed using an unpaired parametric analysis of variance (ANOVA), followed by Tukey’s multiple comparisons test for multiple groups. A value of *p  *< 0.05 was accepted as significant.

## 3. Results

### 3.1. SNA Increased Muscle Weight and Enhanced Grip Strength in DEX-Administered Mice

**Figure 1:**
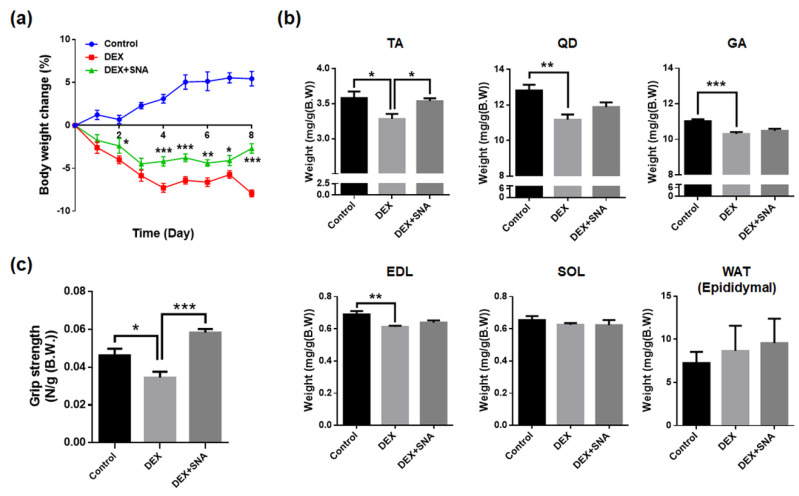
Schisandrin A (SNA) treatment inhibited the loss of body weight and muscle tissue, and increased grip strength in dexamethasone (DEX)-administered mice. C57BL/6 male mice (10 weeks old) were orally treated with SNA (20 mg/kg/day) two days prior to intraperitoneal injection with DEX (20 mg/kg for eight days) and continuously treated for eight days. (**a**) The changes in body weight were monitored daily. Data are shown as mean ± S.E.M., n = 10/group; * *p* < 0.05, * *p* < 0.01, *** *p* < 0.001 vs. DEX. (**b**) Mice were sacrificed, and the tibialis anterior (TA), quadriceps femoris (QD), gastrocnemius (GA), extensor digitorum longus (EDL), and soleus (SOL) muscles, as well as epididymal white adipose tissue (WAT), were carefully excised and weighed. (**c**) Forelimb grip strength tests were performed after 10 days of SNA treatment. Data are shown as mean ± S.E.M., n = 10/group; * *p* < 0.05, ** *p* < 0.01, *** *p* < 0.001.

To investigate the effects of SNA on DEX-induced muscle atrophy, 10-week-old C57BL/6 male mice were orally administered SNA for a total of 10 days, i.e., (20 mg/kg), two days before the administration of DEX (20 mg/kg, i.p. daily for 8 days). As shown in Figure 1a, DEX administration reduced body weight. However, SNA treatment (DEX+SNA group) significantly inhibited this reduction in body weight. We measured the weight of each type of muscle tissue, including the TA, QD, GA, EDL, and SOL muscles, as well as epididymal adipose tissues. DEX administration significantly decreased the muscle tissue weight of TA, QD, GA, and EDL muscles in the DEX group compared to the CON group. The weight of all of these muscle tissues appeared to be increased on SNA treatment, most significantly in the TA muscle, compared with that of the DEX group (Figure 1b). However, the weight of epididymal adipose tissues did not significantly differ among the three experimental groups (Figure 1b). To investigate whether SNA treatment improved muscle function, we compared the forelimb grip strength after 10 days of treatment with SNA. The grip strength was significantly reduced in the DEX group; comparatively, the grip strength was significantly increased in the DEX+SNA group (Figure 1c).

### 3.2. SNA Increased the Muscle Fiber Size in the DEX-Administered Mice

**Figure 2:**
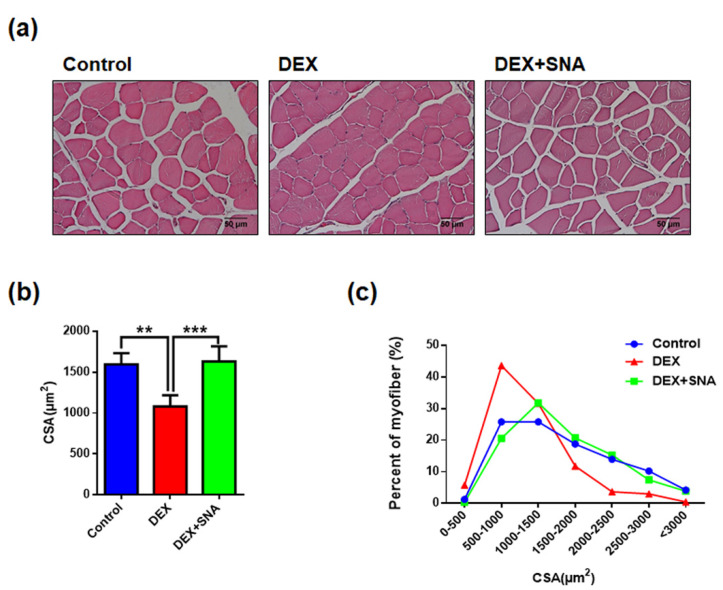
SNA treatment increased the mean muscle myofiber size in DEX-administered mice. The sections from the TA muscle tissue were stained with H&E and observed under a microscope. (**a**) A representative picture is shown. (**b**) The cross-sectional area was measured using ImageJ program and the mean CSA has been represented. (**c**) Muscle fiber size distribution. n = 5/group. Data are shown as mean ± S.E.M., ** *p* < 0.01, *** *p* < 0.001.

The CSA of myofibers was reported to be reduced in dexamethasone-induced muscle atrophy [21]. Therefore, we evaluated if SNA treatment affected the size of the myofibers. TA muscle sections was stained with H&E and the size of myofibers were analyzed. As shown in Figure 2a,b, approximately 30% reduction in mean CSA was observed in the DEX group compared with the CON group. Treatment with SNA significantly increased the CSA compared to the DEX group. In terms of myofiber distribution, the size of myofibers in the ranges of 500–1500 and 1500–3000 µm^2^ in the CON group was approximately 51.6% and 42.8%, respectively, and their proportions changed to approximately 75.2% and 18.2% upon DEX administration. In the DEX+SNA group, the myofiber in the ranges of 500–1500 and 1500–3000 µm^2^ was approximately 52.3% and 43.4%, respectively (Figure 2c). This reduction in mean myofiber size shows that DEX-induced muscle atrophy was significantly inhibited by SNA treatment.

### 3.3. SNA Inhibited the Expression of Muscle Degradation Factors in the TA Muscles of DEX-Administered Mice

**Figure 3:**
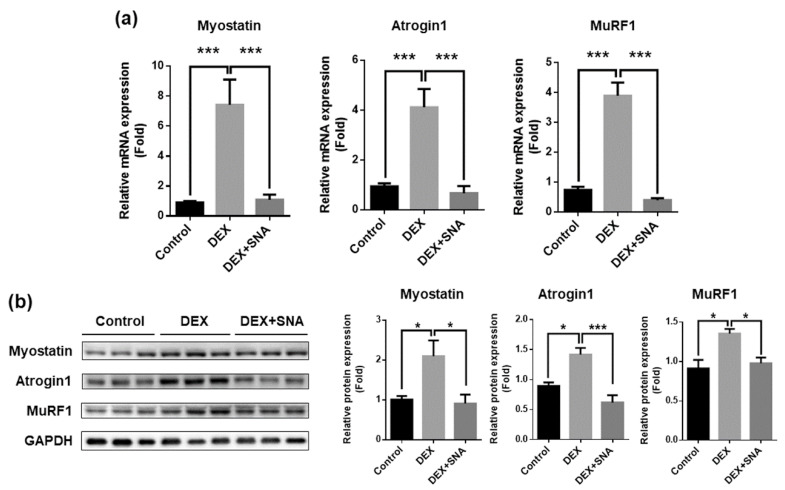
SNA decreased the expression of muscle atrophy factors in DEX-administered mice. (**a**) mRNA expression of myostatin, atrogin1, and MuRF1 in the TA muscle was assessed by RT-qPCR. Cyclophilin B was used as an internal control. (**b**) Protein expression in the TA muscle was evaluated by immunoblotting. The representative blot images have been shown (left panel). The levels of the proteins quantified using ImageJ software and were normalized to GAPDH (right panel). Data are shown as mean ± S.E.M., n = 8 for RT-qPCR, n = 5 for western blot, * *p* < 0.05, *** *p* < 0.001.

To examine if SNA treatment affected the expression of muscle atrophy factors, we analyzed mRNA and protein expression of myostatin, MuRF1, and atrogin1 by RT-qPCR and western blot (Figure 3a,b, respectively). The expression of myostatin mRNA and protein levels were increased in the TA muscle of the DEX group. However, this increase was found to be significantly reduced by SNA treatment in the DEX+SNA group. The expression of two muscle atrophy markers, MuRF1 and atrogin1, was then analyzed. mRNA and protein expression of MuRF1 and atrogin1 was significantly increased in DEX-administered mice and SNA treatment significantly attenuated the expression of these factors.

### 3.4. SNA Decreased the Expression of Muscle Degradation Factors in C2C12 Myotubes

**Figure 4:**
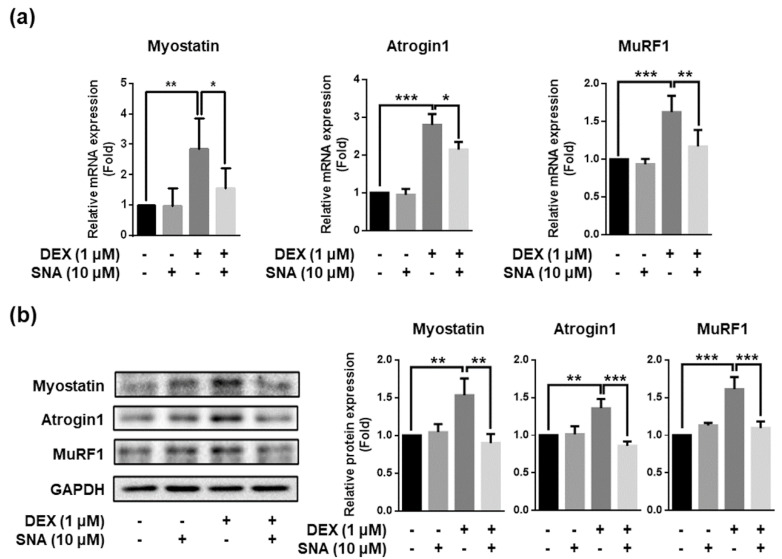
SNA treatment decreased the expression of muscle degradation factors in C2C12 myotubes. C2C12 myotubes were treated with DEX and SNA for 12 h and mRNA and protein levels of myostatin, atrogin1, and MuRF1 were assessed by (**a**) RT-qPCR and (**b**) western blot, Cyclophilin B was used as an internal control for RT-qPCR. The representative blot images have been shown (b; left panel). The levels of the proteins quantified using ImageJ software and were normalized to GAPDH (b; right panel). Data are shown as mean ± S.D., n = 3–4, * *p* < 0.05, ** *p* < 0.01, *** *p* < 0.001.

To confirm the effect of SNA on the expression of myostatin, atrogin1, and MuRF1 in vitro, we treated C2C12 myotubes with 10 µM SNA in the presence of DEX for 12 h. Myostatin, atrogin1, and MuRF1 mRNA and protein expression were then examined. SNA treatment significantly inhibited DEX-induced myostatin, atrogin1, and MuRF1 mRNA and protein expression (Figure 4a,b).

### 3.5. SNA Increased mRNA and Protein Expression of the Myosin Heavy Chain (MyHC) in DEX-Administered Mice and in C2C12 Myotubes

**Figure 5:**
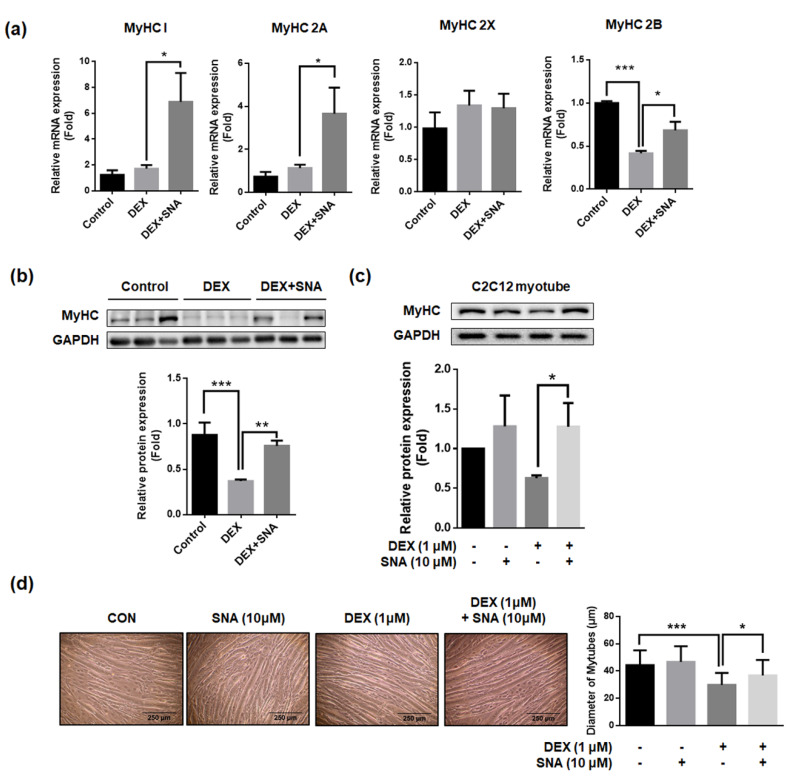
SNA increased the expression of the myosin heavy chain in DEX-administered mice and in C2C12 myotubes. (**a**) The mRNA expression of MyHC 1, 2A, 2X, and 2B in the TA muscle was assessed by RT-qPCR. Cyclophilin B was used as an internal control. (**b**) Protein expression of MyHC in the TA muscle was evaluated by immunoblotting. The representative blot images have been shown (upper panel). The levels of the proteins quantified using ImageJ software and were normalized to GAPDH (lower panel). Data are shown as mean ± S.E.M, n = 4-6 for RT-qPCR, n = 9 for western blot. (**c**,**d**) C2C12 myotubes were treated with 10 μM SNA in the presence of 1 μM DEX for 12 h. (**c**) MyHC expression was detected by western blot. The representative blot images have been shown (upper panel). The levels of the proteins quantified using ImageJ software and were normalized to GAPDH (lower panel). (**d**) The diameter of myotubes was analyzed using ImageJ software. Data are shown as mean ± S.D., n = 3. * *p* < 0.05, ** *p* < 0.01, *** *p* < 0.001.

MyHC is a major component of contractile proteins which acts as a motor protein in muscle tissues [22]. Previously, MyHCs have been associated with muscle thickness, muscle contractile speed, endurance, and strength [22,23,24]. Therefore, the mRNA expression of four isoforms of MyHC (MyHC 1, 2A, 2X, and 2B) were assessed in the TA muscles of the mice (Figure 5a). DEX-administration did not change mRNA expression of MyHC 1, 2A, and 2X, but significantly decreased MyHC 2B mRNA expression. SNA treatment significantly increased the mRNA expression of MyHC 1, 2A, and 2B compared with that in DEX-administered mice. When we assessed the MyHC protein expression in the TA muscle, protein levels were found to have decreased in the DEX-administered mice, and this reduction was significantly inhibited by SNA treatment (Figure 5b). Similarly, we found that the MyHC protein expression (Figure 5c) and myotube diameter (Figure 5d) decreased upon DEX treatment and SNA treatment increased MyHC protein expression in DEX-treated C2C12 myotubes.

### 3.6. SNA Increased the Protein Synthesis and Expression of pAkt, pFoxO, and p70S6K in C2C12 Myotubes

**Figure 6:**
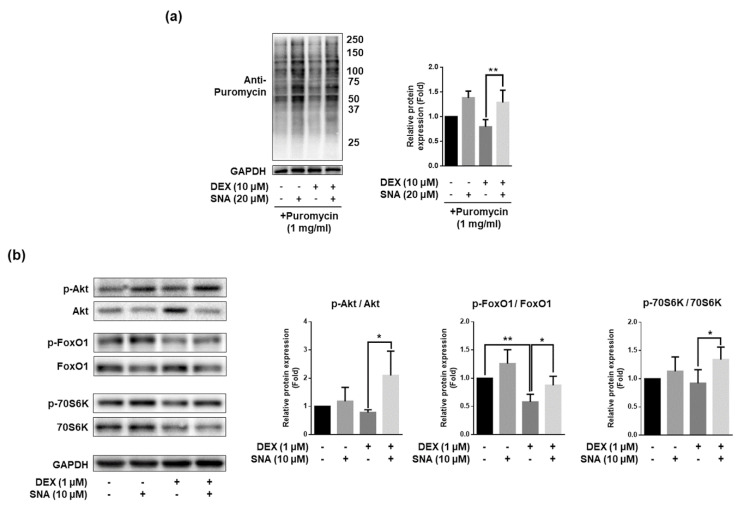
SNA treatment increased protein synthesis and expression of pAkt, pFoxO, and p70S6K in C2C12 myotubes. C2C12 myotubes were treated with 20 μM SNA in the presence of 10 μM DEX for 12 h. (**a**) The puromycin-labelled protein levels were detected by western blot. The representative blot image has been shown (**left panel**). The levels of the proteins quantified using ImageJ software and were normalized to GAPDH (**right panel**), n = 4. (**b**) Protein expression levels of p-Akt, Akt, p-FoxO1, FoxO1, p-70S6K, and 70S6K were assessed by western blot. Data are shown as mean ± S.D., n = 3. * *p* < 0.05, ** *p* < 0.01.

Aside from protein degradation, protein synthesis is also important for the maintenance of skeletal muscle mass [3]. To investigate the effects of SNA, the rate of protein synthesis was analyzed by puromycin incorporation assay (SUnSET assay). DEX treatment significantly decreased the puromycin incorporation and treatment with SNA inhibited this effect of DEX (Figure 6a). It is known that the Akt/FoxO pathway regulates *MuRF-1* expression. Active Akt inhibits FoxO activity, which in turn impedes protein degradation by inhibition of MuRF-1 expression [25]. The Akt/mTOR/p70S6K pathway increases protein synthesis, and thus promotes hypertrophy [25]. Therefore, we investigated the expression of these signaling molecules and found that the expression of phosphorylated Akt, FoxO1, and 70S6K was decreased by DEX treatment. SNA treatment reversed the effects of DEX (Figure 6b).

## 4. Discussion

The fruits of *S. chinensis* have been used in traditional medicine in Korea and China and are known to be effective in the treatment of the cardiovascular diseases, fatigue and weakness, and insomnia [17,26]. Previously, we found that the ethanolic extract of the Schisandra fruit significantly improved muscle strength and increased muscle mass in aged mice [27]. In addition, the extract significantly downregulated myostatin expression in muscle tissues [8,27]. However, the bioactive component of Schisandra responsible for this biological activity remains undetermined. The extract from the fruits of *S. chinensis* contains various bioactive components, including lignans [17]. We evaluated the effect of several lignans on the expression of myostatin, a major factor affecting muscle degradation, and found that SNA was most effective in down-regulating myostatin (data not shown) and was thus used for further analyses.

We discovered that SNA prevented DEX-induced muscle atrophy in mice and increased muscle function and muscle weight. SNA has been reported to have anti-oxidant, anti-inflammatory, anti-cancer, anti-viral, and hepatoprotective effects [17,28,29,30]. SNA supplementation improves the condition of nonalcoholic fatty liver disease in mice that were fed a high-fat/high-cholesterol diet [30]. Further, SNA suppresses inflammation, apoptosis, and oxidative stress by suppressing the PI3K/Akt, MAPK, and NF-κB signaling pathways in various cell types [28,31] and activates the Nrf2/HO-1 or AMPK/Nrf2 pathway [32,33]. Moreover, SNA prevents ROS-induced DNA damage and apoptosis in C2C12 cells [29]. The anti-inflammatory and anti-oxidative effects of SNA may be beneficial for the treatment of muscle atrophy because inflammation and oxidative stress contribute to muscle wasting [34,35].

DEX belongs to the family of GCs and is used as an immunosuppressive drug [36]. However, prolonged treatment with high doses of DEX causes muscle atrophy by decreasing muscle turnover and increasing muscle degradation [8]. In the body, GC levels are known to increase during fasting, sepsis, and aging [37,38,39]. An increase in GC levels could promote the expression of genes related to protein degradation. We found that DEX administration led to a significant reduction of grip strength in mice, while SNA treatment significantly increased it. This suggested that SNA prevented the decrease in muscle function induced by DEX. We found that the weights of the TA, EDL, GA, and QD muscles, except of SOL, were reduced in mice administered with DEX. Treatment with SNA significantly inhibited the reduction of TA muscle weight (Figure 1b). The composition of each type of myofiber (MyHC 1, 2A, 2X, and 2B) in TA, SOL, EDL, GA, and QD muscles is different. The SOL muscle is mainly composed of slow fibers (MyHC 1 or 2A). DEX-induced muscle atrophy causes a decrease in fast muscle fibers [40]. This may explain why the muscle weight of SOL was not affected by DEX.

Reduced muscle function is associated with the decrease in the size of muscle fibers [41]. Analysis of the internal structure of muscle fibers showed that the mean CSA of TA muscles in mice administered with DEX was reduced, and the average CSA showed smaller fibers (Figure 2c). Conversely, treatment with SNA inhibited the reduction of CSA and reversed this effect. These results indicate that SNA treatment increases muscle fiber size, contributing to reduced muscle wasting and enhanced muscle strength.

Myostatin is a negative regulator of muscle mass and a key factor determining the size and number of muscle fibers. It is known to induce muscle atrophy in various conditions and is thus a key target for the treatment of muscle atrophy. The in vivo and in vitro analyses showed that myostatin expression was increased by DEX, and this increase was inhibited by SNA. DEX also promotes the expression of protein degradation factors via other pathways, one of which is through altering KLF15 expression [7]. In our study, the expression of KLF15 was increased by DEX but was not affected by SNA treatment (data not shown). Therefore, SNA treatment might attenuate muscle atrophy through the downregulation of the myostatin pathway. Muscle protein degradation is promoted by the UPS, caspase system pathway, and autophagy pathway [4]. UPS is a key pathway which contributes to the loss of muscle [42]. Atrogin1 and MuRF1 are two major muscle proteins related to the UPS signaling pathway and play an important role in the degradation of contractile proteins [43]. The expression of atrogin1 and MuRF1 was increased by DEX, and SNA treatment inhibited this increase both in vivo and in vitro (Figure 3 and Figure 4), reducing the muscle protein degradation. When we examined the Akt/FoxO pathway, which regulates the expression of MuRF1 [25], phosphorylation of FoxO1 was increased in DEX- and SNA-treated C2C12 myotubes compared to that of DEX-treated myotubes (Figure 6b). These results indicate that SNA could decrease the expression level of MuRF1 by inhibiting FoxO activity.

MyHC, a major component of contractile proteins, acts as a motor protein in muscle tissues and is related to the muscle thickness, muscle contractile speed, endurance, and strength [22,23,24]. Fast myofiber is known to be decreased in DEX-induced muscle atrophy mice [44]. Corresponding with this result, we found that mRNA expression of MyHC 2B, a fast fiber isoform, was decreased in TA muscles after DEX administration. However, mRNA expression of slow fiber isoforms, MyHC 1 and 2A, did not change upon DEX administration. SNA increased mRNA expression of both slow and fast myofiber isoforms (MyHC 1, 2A, and 2B) (Figure 5a). Further, the expression of total MyHC proteins was reduced by DEX, and SNA was able to reverse this effect (Figure 5b,c). In C57BL/6 mice, because the TA muscles contain a higher percentage of slow fibers (MyHC 1 and 2A) compared to the QD and GA muscles, the increase in MyHC 2A mRNA expression by SNA treatment might be much greater than that of MyHC 2B (Figure 5a).

Skeletal muscle atrophy is induced not only by increased protein degradation but also by decreased protein synthesis. It was reported that DEX treatment resulted in reduced protein synthesis by inhibition of the Akt / mTOR pathway [45]. We found that DEX decreased the levels of puromycin-incorporated proteins and that treatment with SNA was able to reverse this effect, even in the presence of DEX. In addition, the expression of phosphorylated Akt/70S6K was increased in DEX- and SNA-treated C2C12 myotubes compared with that in DEX-treated myotubes (Figure 6b). These results suggest that SNA treatment increased protein synthesis through the Akt/70S6K pathway and MyHC protein expression, thereby reducing muscle wasting.

## 5. Conclusion

In this study, we found that SNA treatment increased the muscle mass and grip strength in DEX-induced muscle atrophy in mice by blocking protein degradation and stimulating protein synthesis. SNA treatment inhibited the expression of atrogin1, MuRF1, and myostatin which were induced by DEX administration, both in vivo and in vitro. In addition, SNA treatment increased the expression of MyHC and the protein synthesis. Although we found that Akt/FoxO and Akt/70S6K pathways are involved, further studies are needed to understand the detailed molecular mechanisms involved in inhibiting the expression of protein degradation factors by SNA and its influence on the signaling pathways to increase protein synthesis.

## Figures and Tables

**Table 1:** Primer sets used for quantitative PCR analyses.

No.	Primer	Sense	Anti-Sense
1	MuRF1	AGGACTCCTGCAGAGTGACCAA	TTCTCGTCCAGGATGGCGTA
2	Atrogin1	GCAAACACTGCCACATTCTCTC	CTTGAGGGGAAAGTGAGACG
3	Myostatin	GGCCATGATCTTGCTGTAA	TTGGGTGCGATAATCCAGTC
4	MyHC * 1	CCAAGGGCCTGAATGAGGAG	GCAAAGGCTCCAGGTCTGAG
5	MyHC * 2A	AAGCGAAGAGTAAGGCTGTC	GTGATTGCTTGCAAAGGAAC
6	MyHC * 2X	CACCGTCTGGATGAGGCTGA	TGTTTGCGCAGACCCTTGATAG
7	MyHC * 2B	ACAAGCTGCGGGTGAAGAGC	CAGGACAGTGACAAAGAACG
8	Cyclophilin B	TGGAGAGCACCAAGACAGACA	TGCCGGAGTCGACAATGAT

* MyHC = myosin heavy chain.

## Data Availability

The data used to support the findings of this study are available from the corresponding author upon request.
